# Female and male Eurasian lynx have distinct spatial tactics at different life‐history stages in a high‐density population

**DOI:** 10.1002/ece3.7846

**Published:** 2021-06-29

**Authors:** Deniz Mengüllüoğlu, Sarah Edwards, Heribert Hofer, Anne Berger

**Affiliations:** ^1^ Leibniz Institute for Zoo and Wildlife Research (IZW) Berlin Germany; ^2^ Department of Biology, Chemistry, Pharmacy Freie Universität Berlin Berlin Germany; ^3^ Capel Manor College Waltham Cross, Enfield UK; ^4^ Department of Veterinary Medicine Freie Universität Berlin Berlin Germany

**Keywords:** floaters, GPS locations, home range, population density, spatial capture–recapture, spatial tactics, territoriality

## Abstract

Knowledge regarding the spatial behavior of the Eurasian lynx is mainly inferred from populations in Europe. We used GPS telemetry to record the spatial behavior of nine individuals in northwestern Anatolia obtaining eleven home ranges (HRs). Analyses revealed the smallest mean HR sizes (n_HR_ **_♀_** = 4) at 57 km^2^ (95% kernel utilization distribution, KUD) and 56 km^2^ (95% minimum convex polygon, MCP), ever reported for adult female Eurasian lynx. Adult males either occupied small permanent territories (n_HR♂_._T_ = 2), with a mean of 176 km^2^ (95% KUD) and 150 km^2^ (95% MCP), or were residents without territories (floaters, n_HR♂_.*_F_* = 2) roaming across large, stable HRs with a mean size of 2,419 km^2^ (95% KUD) and 1,888 km^2^ (95% MCP), comparable to HR sizes of Scandinavian lynx populations. Three disperser subadult males did not hold stable HRs (mean 95% KUD = 203 km^2^, mean 95% MCP = 272 km^2^). At 4.9 individuals per 100 km^2^, population density was one of the highest recorded, suggesting that the presence of adult male floaters was a consequence of a landscape fully occupied by territorials and revealing a flexibility of spatial behavior of Eurasian lynx not previously recognized. Such a high population density, small HRs, and behavioral flexibility may have been aided by the legal protection from and apparent low levels of poaching of this population. The observed spatial tactics are unlikely to be seen in most of the previously studied Eurasian lynx populations, as they either suffer medium to high levels of human‐caused mortality or were unlikely to be at carrying capacity. For effective and appropriate conservation planning, data from felid populations in a reasonably natural state such as ours, where space, density, prey, and pathogens are likely to be the key drivers of spatial dynamics, are therefore essential.

## Graphical abstract

In this study, we report the home range (HR) size, spatial behavior, and tactics of Eurasian lynx L. l. dinniki using high frequency GPS tracking data and focusing on the potentially isolated yet unexploited northwest Anatolian population. We also consider the HR size and density of L. l. dinniki in the context of previously published data for autochthonous and reintroduced Eurasian lynx populations in Europe (subspecies L. l. lynx and L. l. carpathicus).

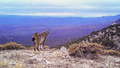

## 1 INTRODUCTION

Many carnivores are solitary (Gittleman, 1989), with males and females maintaining independent home ranges (HRs). This is largely the case in felids, where only two species, the lion (*Panthera leo*) and the domestic cat (*Felis catus*), have been recognized to be social (Macdonald et al., 2000; Schaller, 1972). Detailed studies on cheetahs (*Acinonyx jubatus*) demonstrated that even in felids with a simple social organization, complex male spatial tactics can be observed which include both territorial residents and resident floaters, representing different life‐history stages of adult males (Caro, 1994; Melzheimer et al., 2018, 2020). In the cheetah populations in the Serengeti and Namibia, where these complex male spatial tactics were observed, sites for male territories were limited and floaters usually waited for a vacancy to arise (Melzheimer et al., 2018, 2020). Such a queueing system is a form of organization whose importance is only being gradually recognized and which poses interesting evolutionary questions (e.g., Maynard Smith, 1982). Some male queues have been described for mammals such as male mating queues in thirteen‐lined ground squirrels *Ictidomys tridecemlineatus* (Schwagmeyer & Parker, 1987), male queues for harem territories in greater sac‐winged bats *Saccopteryx bilineata* (Voigt & Streich, 2003), male social queues for dominance rank in the spotted hyena *Crocuta crocuta* (East & Hofer, 2001), and offspring queuing to inherit parental territories in the red fox *Vulpes vulpes* (Lindström, 1986). The cheetah studies suggest that populations of solitary felids at, or close to carrying capacity, could develop a queueing system for territory ownership in males. Similar to the floaters and territorials observed in cheetahs, queueing and territory ownership might represent different life‐history stages of adult males in other carnivores (the high population density hypothesis).

The Eurasian lynx *Lynx lynx* population in northwest Anatolia was previously reported to display high genetic diversity and no signs of inbreeding, suggesting that this lynx population did not suffer a recent bottleneck (Mengüllüoğlu et al., 2019). All lynx populations in Turkey are legally protected, no hunting quotas are issued and the populations are apparently little poached, although poaching is known to take place in some parts of Anatolia (Şekercioğlu et al., 2011). Lynx populations in Anatolia can occur at high densities (4.2 independent lynx/100 km^2^ Avgan et al., 2014), and many territorial individuals occur in a small area (Mengüllüoğlu et al., 2019). Therefore, the northwest Anatolian lynx population is an example of a nonexploited population likely to display behavior in terms of spatial tactics (such as queuing for territories by floaters), individual interactions and associated population dynamics typical for natural populations at high densities and at or close to carrying capacity (Caro, 1994; Melzheimer et al., 2018).

**FIGURE 1:**
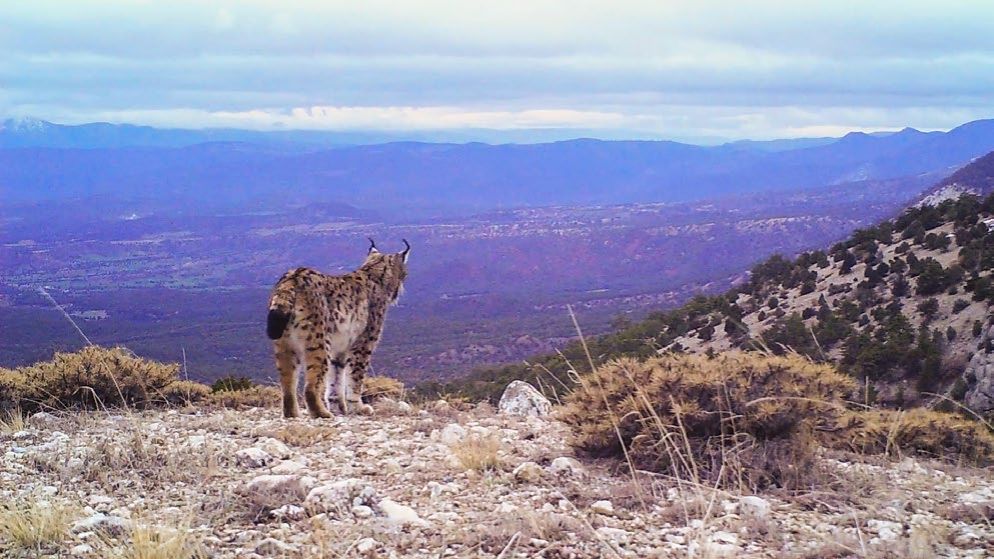
A camera trap picture of a territorial male Eurasian lynx *Lynx lynx dinniki* in its xeric coniferous habitat in Nallıhan Mountains, northwestern Anatolia, Turkey (photo: Deniz Mengüllüoğlu & Nurten Şalıkara)

The spatial ecology and organization of Eurasian lynx in central and northern Europe have been widely studied through radio‐telemetry. Some of these studies reported the largest known mean territorial HR sizes for felids (Herfindal et al., 2005; Linnell et al., 2001, 2021), recorded at northern latitudes. In central and Eastern Europe HR size generally decreased toward southern latitudes while simultaneously environmental productivity increased (Herfindal et al. 2005; but: Schmidt et al., 1997). Herfindal et al., (2005) suggested that environmental productivity reflected prey density and that this was the main driver of HR size in Eurasian lynx populations (the primary production hypothesis). In most of Anatolia, the Eurasian lynx (*L.l. dinniki*) occurs in xeric coniferous forests, open steppe habitats with scattered trees, and open rocky habitats distributed over mountains and plateaus (Figure 1; Mengüllüoğlu et al., 2018). These ecosystems generally exhibit lower primary production than north Anatolian (Black Sea coast) or central European humid and temperate mixed and deciduous forests (Evrendilek et al., 2007). A recent study of ten study sites in northern Anatolia reported that lynx presence was significantly positively correlated with brown hare *Lepus europaeus* presence and the presence of coniferous woodland rather than the presence of roe deer *Capreolus capreolus* and the type of humid deciduous habitat which roe deer occupy in Turkey (Soyumert et al., 2019). Therefore, the primary production hypothesis (Herfindal et al., 2005) predicts lower lynx densities and larger HR sizes for lynx populations in Anatolia than in central Europe because Anatolian lynx appears to be associated with less productive habitats.

Therefore, if the high‐density hypothesis was driving the spatial behavior of Eurasian lynx in Anatolia, we expected territorial lynx to maintain relatively small HRs. We also expected to observe floater individuals roaming over large areas in queue for territory ownership. Alternatively, if the primary production hypothesis was driving the spatial behavior of Eurasian lynx in Anatolia, we expected the population to be at relatively low density, that territorial lynx would maintain large HRs, and we would not observe adult floater individuals. In order to test these predictions from the “high density” and the “primary production” hypotheses, we studied lynx in northwestern Anatolia at a site where we have been monitoring Eurasian lynx through camera trapping since 2009. We used camera trap data to assess lynx density and used GPS telemetry to observe the spatial behavior of male and female adult lynx, and record their movements and sizes of their HRs.

## 2 METHODOLOGY

### 2.1 Study area

**FIGURE 2:**
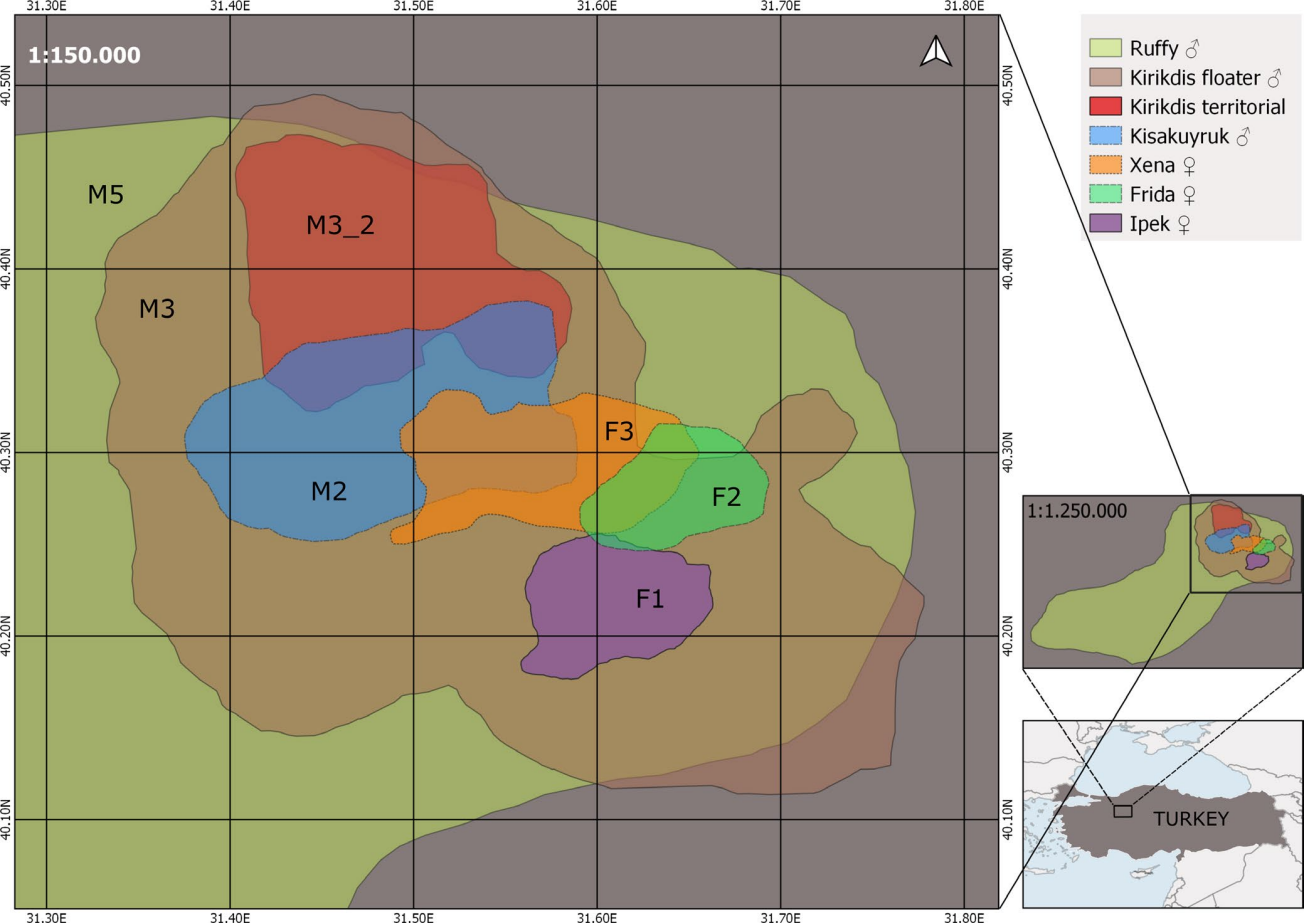
Location of the study area in the Nallıhan Mountains in Turkey and 95% kernel utilization distribution home ranges of resident adult Eurasian lynx *Lynx lynx* tracked between 2015 and 2017. The home ranges of one individual male, M3 are presented separately for the floater and territorial stage. Produced using Quantum GIS (2015)

We conducted the study in Nallıhan Mountains (400 km^2^; 40°11′–31°21′; Figures 1 & 2), a mountain chain in the transition zone between the dry western Black Sea (xero‐euxine) and central Anatolian (Iran–Turan) floristic zones in Turkey. This region is also influenced by the Mediterranean floristic zone (western Aegean), through the catchment area of the Sakarya River (Aksoy, 2009). Vegetation and landscape have been shaped by altitude and historical human use. The lower areas (500–1,000 m) are covered by steppe in the south, gradually replaced by xeric coniferous forests up to 1,500 m which are composed of black pine *Pinus nigra* and junipers (*Juniperus excelsa* and *J. oxycedrus*) with an understory of oak‐dominated scrub (*Quercus pubescens, Pyrus elaeagnifolia, Crataegus* spp.) with frequent forest openings. Mean annual temperature is 12.2°C, with minimum and maximum temperatures of −18.8°C in January and 40.2°C in August, respectively (1975–2010 statistics; mgm.gov.tr). Mean annual total precipitation was 308.7 mm, with highest and lowest mean monthly precipitations of 42.6 mm in January and 8.1 mm in August, respectively (1975–2010 statistics; mgm.gov.tr). The study area does not hold any form of protection status and is part of the state forests management system. The human population in this area is at a low density and restricted to several villages in the surrounding lowland and valleys. Red deer *Cervus elaphus* and wild boar *Sus scrofa* are the common large herbivores, and brown hare is the main lynx prey species (Mengüllüoğlu et al., 2018). At higher elevations, brown bear *Ursus arctos* and gray wolf *Canis lupus* are sympatric with the lynx. Golden jackal *Canis aureus*, red fox, and jungle cat *Felis chaus* occur at lower elevations and rarely in the habitat occupied by lynx and wolves (Mengüllüoğlu, 2010).

### 2.2 Live trapping and tracking

We performed live trapping in collaboration with the Wildlife Department of the Turkish Ministry of Agriculture and Forestry (WDT) under protocol and permit number 30057506‐030‐1867. We used five cage traps made of metal mesh, produced by the WDT (length: 2 m, height: 1.5 m, width: 1 m) that were placed on lynx trails at nine live trapping stations throughout the capture surveys. We monitored the cage traps continuously via very high frequency (VHF) transmitters and General Packed Radio Service (GPRS) camera traps (KeepGuard KG860, Keeptime industrial (Asia) Co., Hong Kong, China) that send pictures of the cage trap once an animal enters. We also visited and checked each trap every second day. Over the course of three trapping seasons (*n* = 961 active trap days) during the winters of 2014, 2015, and 2016 from December until April in the following year, we captured 16 lynx (five females and 11 males).

We estimated lynx age on the basis of tooth wear (Table 2 in Marti & Ryser‐Degiorgis, 2018). We fitted nine lynx individuals with 185 g GPS collars (e‐obs GmbH, Grünwald, Germany) after anesthesia at a dose of 5 mg/kg ketamine and 0.2 mg/kg medetomidine injected (for assumed mean body masses of 10 kg for juveniles; Breitenmoser et al., 1993 and 15 kg for adults; WDT, unpublished data) by the help of a blowpipe. After taking morphometric measurements and fitting GPS collars, we applied a dose of 0.2 mg/kg atipamezole to reverse the sedative effect of medetomidine. Anesthesia was carried out by the authorized wildlife veterinarian of the WDT according to national ethical legislation. No specific permit was required for anesthesia and handling of lynx as it was conducted by the WDT. We did not anesthetize or collar one old adult male lynx captured in 2015 (who had been regularly recorded during continuous camera trap monitoring since 2009) and two 6‐month‐old kittens captured in 2016 because of ethical concerns regarding the suitability of their age for anesthesia. Four other adult lynx, three males and one female, trapped during the first two live trapping periods escaped as the traps produced by the WDT were still under development and had some weak sides which were subsequently strengthened. Rate of live capture was 1 lynx per 60 trap days for all captured lynx and 1 lynx per 107 trap days for the collared lynx.

The GPS collars recorded GPS data ranging between 12 and 21 locations per day. We tracked the lynx on a mean of 250 ± 224 days (*SD*, range 19–612) and obtained on average 4,154 ± 3,926 GPS locations (*SD*, range 285–11,173, Table 1) per lynx. We downloaded GPS data via handheld UHF antennas from hilltops. We could track one 11‐month‐old male (M4) and one 4‐year‐old resident female (F2) at an average of 20 days whence the GPS collars failed. One 2‐year‐old male (M6) was tracked for 20 days and we could not gather data after dispersal from the study area. Locating GPS‐collared lynx and downloading data via handheld antenna (e‐obs GmbH, Grünwald, Germany) was difficult because of the rugged montane topography of the study area cut by many valleys and series of heights.

**TABLE 1:** Total distance moved (TDM), total number of GPS fixes (GPS), tracking days (T), daily distance moved (DDM), and mean number of daily GPS fixes (mean GPS) per tracked Eurasian lynx individual in NW Anatolia between 2015 and 2017

Name	Sex/ID	TDM (km)	GPS	T (days)	DDM (km)	Mean GPS
Frida	F2	79	276	20	4.0	13.8
Ipek 2015	F1	1889	7,256	365	5.2	19.9
Ipek 2016	F1_2	1,145	3,915	197	5.8	19.9
Xena	F3	1,401	4,591	241	5.8	19.0
Evrim	M4	85	300	18	4.7	16.7
Finger	M6	126	284	20	6.3	14.2
Kirikdis 2016	M3	2069	4,811	365	5.7	13.2
Kirikdis 2017	M3_2	1,435	3,176	246	5.8	12.9
Kisakuyruk	M2	851	2081	160	5.3	13.0
Ruffy	M5	1,378	3,459	262	5.3	13.2
Uluhan	M1	1756	7,232	345	5.1	21.0
Mean all lynx			3,398.3	203.5	5.4	16.1
*SD*			2,531.8	135.1	0.6	3.3
Mean males			3,049.0	202.3	5.5	14.9
*SD*			2,480.8	142.2	0.5	3.0
Mean females			4,009.5	205.8	5.2	18.2
*SD*			2,876.7	142.8	0.9	2.9

Note

For lynx with more than one year of tracking data (F1 and M3), results are given separately per tracking year.

We calculated the individual and mean minimum daily distance moved (DDM) separately for males, females and for all collared lynx by dividing total distance moved by the number of tracking days. For individuals with data for more than one year, we calculated DDM for each year. As the GPS collars recorded a minimum of 12 and a maximum of 21 GPS fixes per day, we assumed the collected data were reliable to estimate the DDM per lynx individual. We also compared the body masses of northwest Anatolian lynx with those from other studies using nonparametric Mann–Whitney *U* test (Hollander et al., 2014). We performed the statistical tests in PAST software (Hammer et al., 2001).

### 2.3 Home range analysis

We estimated HR sizes with 95% and 50% kernel utilization distributions (KUD) and 100%, 95%, and 50% minimum convex polygons (MCP) using the R (R Core Team, 2019) package “*adehabitatHR*” version 0.4.16 (Calenge, 2011). We classified the lynx individuals into adult males, adult females, and subadult males on the basis of body size, tooth wear, and camera trap monitoring since 2009. If an individual was frequently photographed by particular camera traps (from our continuous camera trapping since 2009) and breeding in the study area (i.e., breeding females) prior to live capture and collaring, it was classified as resident territorial. All results are presented as means ± standard deviations.

For individuals with data for more than one year (F1 and M3), we calculated separate HRs for each year. For a resident territorial breeding female lynx (F2) which had less than one month of tracking data, we tested whether the collected data were sufficient for reliably estimating its HR. Hence, for each female lynx (F1‐F3), we calculated cumulative MCP HR sizes for each day passed after the first location (Laver & Kelly, 2008) using all locations or a single random location per day (Melovski et al., 2020). We also compared the first 35 days of 95% KUD HR of an adult female lynx (F1) monitored for a long period with its annual 95% KUD HR. In the comparison, the breeding and denning period (15 April 2015 to 14 September 2015) were excluded from the annual HR of the long‐term monitored female lynx as during breeding and denning female HRs are smaller than their HR during other seasons (Schmidt et al., 1997). The 21 days HR of the short‐term monitored female lynx (F2) did not overlap with this period. Hence, we compared 5th, 8th, 10th, 15th, 20th, 25th, 30th, and 35th days’ 95% KUD HR size to mean 95% KUD HR size of mating, autumn, and winter seasons, but the duration between breeding and Autumn was discarded. We did not implement this method for two young male lynx (M4 and M6) with short‐term tracking data as they did not have stable territories.

### 2.4 Capture–mark–recapture (CMR) survey

We conducted the camera trapping survey at 17 camera trap stations using Reconyx HC600 (Holmen, Wisconsin, USA) and Bushnell Trophy Cam 119678HD MAX (Bushnell Co., Overland Park, Kansas, USA) infrared camera traps, using between two and four cameras per location. Cameras were active 24 hr a day and set to continuously record with no delay between consecutive images or videos. We set some cameras near live trapping stations as the live traps were placed along active lynx trails recognized from long‐term population monitoring. Camera trapping was part of long‐term lynx monitoring, and therefore, cameras were active throughout the year. We chose the period from 24 December 2015 to 02 April 2016 (100 days) to estimate lynx population density in order to capture high lynx activity before and during mating season (Breitenmoser et al., 2006). We used a longer survey period to increase the number of photographic recaptures as some lynx individuals had been trapped in the area already (7 lynx in winters 2014–2015 and 2015–2016) and might have been wary of camera traps (Weingarth et al., 2015). Therefore, we increased the twelve 5‐day occasions generally used in Eurasian lynx CMR studies to twenty 5‐day occasions.

We applied a camera‐trapping study design similar to other Eurasian lynx camera trapping surveys. This meant that the mean nearest neighbor distance between camera traps was 2.2 ± 0.8 km (range 1.2 km to 3.9 km). We did not use a grid system, as camera trapping was designed to serve other purposes (e.g., live trap monitoring) as well as the CMR survey.

### 2.5 Population density estimation

We used the R (R Core Team, 2019) package secr version 3.2.0 (Efford, 2012) to estimate density using a maximum likelihood framework. The package requires three input files. The first, the “capture history” file, was created by individually identifying lynx using unique pelage pattern and assigning sex using the presence or absence of the external scrotum with the testes and the presence of associated kittens. We then constructed individual capture histories using twenty 5‐day occasions (Avgan et al., 2014). The second input file, the “trap deployment” file, details the UTM GPS locations of camera traps, along with a binary string to represent when a particular detector was active (“1”), or inactive (“0”) during a sampling occasion. The third input file, the habitat mask, represents the habitat in the vicinity of the detectors potentially occupied by the species of interest and can delineate habitat and nonhabitat sites within the outer limit (Efford, 2019). We constructed the habitat mask in QGIS 3.6.0 (2015) by placing a 15.44 km buffer around the minimum convex polygon (MCP) of the camera trap locations, and overlaying a shape file layer containing areas of nonhabitat within the buffer area to create a shapefile of the suitable habitat around the camera traps. We used a buffer of 15.44 km as this was the mean maximum distance moved (MMDM) by six GPS‐collared male lynx in the study area. We defined the unsuitable habitat as open agricultural fields and villages around the study area that were not used by the collared lynx individuals (*n* = 9). After removal of the unsuitable habitat, the 15.44 km buffer resulted in a sampling area of 1,048 km^2^.

We ran SCR density models to select the most appropriate detection (observational) process, either half‐normal or negative exponential, using Akaike's information criterion adjusted for small sample size (AICc) for either model. We did not consider the hazard rate detection process, as this is only recommended in situations in which the survey area is fully surrounded by a natural or artificial boundary, given that density estimates from it do not reach a plateau fairly promptly with an increasing buffer width (Efford, 2017). We ran three density models, using the most appropriate detection process, in which g_0_(λ_0_), the capture probability at the center of an individual's HR, and б, a function of the scale of animal movement, were affected by various factors: (1) the null model in which both g_0_ and б were constant (λ_0_ ~ 1, б ~ 1), (2) the behavior b_1_ model in which g_0_ was affected by the response of individuals to camera traps (λ_0_ ~ b, б ~ 1), (3) a second behavior model, the learnt response b_2_, in which both g_0_ and б were affected by the response of individuals to camera traps (λ_0_ ~ b, б ~ b). Due to small sample sizes, sex‐specific models were not considered. We ranked all models using AIC_c_ values. We tested population closure by performing the closure test (Otis et al., 1978) within the secr package. After obtaining the lynx population density in our study area, we investigated the relationship between female HR size and lynx population density using data from eight lynx populations located in a north to south latitudinal gradient. We ran nonparametric correlations using PAST v.4.05 (Hammer et al., 2001).

## 3 RESULTS

### 3.1 Tracked lynx

We captured and collared two breeding adult (F1 and F2) and one nonbreeding adult female lynx (F3), and three adult (M2, M3, and M5), one young adult (M6), and two juvenile male lynx (M1 and M4, Tables 1 and 2). Both young adult lynx (F3 and M6) were 23 months old, and both juveniles were 11 months old at capture time. The other five collared lynx were older than 4 years (Table 2). Mean body mass of adult lynx from both sexes was smaller than masses of adult Eurasian lynx in European populations. Mean body mass of adult female lynx in our study area (13.1 ± 0.4 kg, *n* = 3) was significantly smaller than that of female lynx in Switzerland (17.6 ± 1.9 kg, *n* = 4; Mann–Whitney *U* test, U = 0, *p* = .05; data from Breitenmoser et al., 1993). Adult male mean body mass in our study area (16.6 ± 0.9 kg, *n* = 4) was also significantly smaller than of male lynx in Switzerland (22.0 ± 1.5 kg; U = 0, *p* =.05). Mean adult body masses of both sexes of northern lynx (males = 21.4 ± 2.1, females = 15.4 ± 0.8; Jedrzejewski et al., 1996) and lynx in Siberia (males = 21 ± 0.7; Sedalishchev et al., 2014) were also larger than the mean body masses of Eurasian lynx in Anatolia. There was no significant difference between mean body masses of juvenile lynx from our study area (10.0 ± 0.6 kg, *n* = 2) and juvenile lynx in Switzerland (11.2 ± 1.6 kg; U = 4.5, *p* = .28).

**TABLE 2:** Capture dates, ages and body mass at capture, and individual and mean home range sizes (km^2^) of Eurasian lynx *Lynx lynx* individuals tracked in NW Anatolia between 2015 and 2017

Name	ID	Date of capture	Age at capture	Body mass [kg]	100% MCP [km^2^]	95% MCP [km^2^]	50% MCP[km^2^]	95% KUD [km^2^]	50% KUD [km^2^]
Territorial females									
İpek 2015	F1	20.03.2015	9–10 a	12.8	69.2	55.5	18.0	54.1	18.4
İpek 2016	F1_2				48.9	37.7	10.2	36.7	11.6
Frida	F2	21.03.2017	4–5 a	13.6	30.2	28.0	16.7	44.1	14.2
Xena (nonbreeding	F3	29.03.2017	23 mo	13.0	194.9	101.4	15.5	94.6	15.3
Mean				13.1	85.8	55.7	15.1	57.4	14.9
Territorial adult males									
Kırıkdiş 2017	M3_2				179.5	145.1	40.9	174.0	44.0
Kısakuyruk	M2	08.03.2016	7–8 a	15.6	186.2	155.6	63.9	178.1	45.8
Mean					182.9	150.4	52.4	176.1	44.9
Nonterritorial adult males (floaters)									
Kırıkdiş 2016	M3	24.03.2016	7–8 a	16.5	787.3	703.6	359.3	857.7	222.0
Ruffy	M5	08.03.2017	9–10 a	17.7	3,531.1	3,072.2	1727.0	3,980.6	1,031.9
Mean				16.6*	2,159.2	1887.9	1,043.2	2,419.2	627.0
Nonterritorial young males (dispersers)									
Uluhan	M1	20.03.2015	11 mo	10.6	932.3	713.0	73.5	447.3	68.6
Evrim	M4	17.03.2016	11 mo	9.4	41.7	38.4	10.6	57.0	14.6
Finger	M6	21.03.2017	23 mo	16.5	71.4	65.9	23.3	103.9	29.6
Mean				10.0**	348.5	272.4	35.8	202.7	37.6

Note

Age at capture is given in years (a) or months (mo). Sizes of home ranges are given for minimum convex polygons (MCP) and kernel utilization densities (KUD).

* Calculated using body masses of M2, M3, M5, and M6.

** Calculated using body masses of M1 and M4.

All three collared females held territories, whereas only one adult male lynx (M2) held a territory at the time of capture and collaring. This male individual had been repeatedly captured by our camera traps in the same area since 2012 and stayed in the same area after capture and collaring. Even after the battery of his collar failed camera traps continued to repeatedly capture this individual until July 2018. Two other adult males (M3 and M5) not known prior to capture and collaring were apparently nonterritorial adults and moved across stable but very large HRs. We therefore classified these two males as ‘floaters’. At the second year of GPS tracking, M3 shrank his HR to one‐fifth the size of his HR during the first tracking year (Figure 2). Two juvenile male lynx were born inside and dispersed from the study area (M1, M4), and one subadult male lynx (23 months old, M6) was not encountered before capture in camera trap pictures, captured and collared inside the study area, and left without a re‐encounter event for data downloading. Therefore, we classified these male lynx as “dispersers.” Hence, our study population displayed three spatial behavior categories: adult female and male “territorials with small stable territories,” adult male “floaters with very large and stable HRs,” and young “disperser individuals with expanding HRs.”

Mean DDM by individual lynx ranged from 4.0 to 6.3 km with a mean of 5.4 ± 0.6 km (Table 1). The mean DDM of female lynx (n_lynx_ = 4, n_locations_ = 16,038, m_DDM_ = 5.2 ± 0.9 km) was not significantly different from the mean DDM of male lynx (*n* = 7, n_locations_ = 21,343, m_DDM_ = 5.5 ± 0.5 km; U = 12, *p* = .74).

### 3.2 Home range size

**FIGURE 3:**
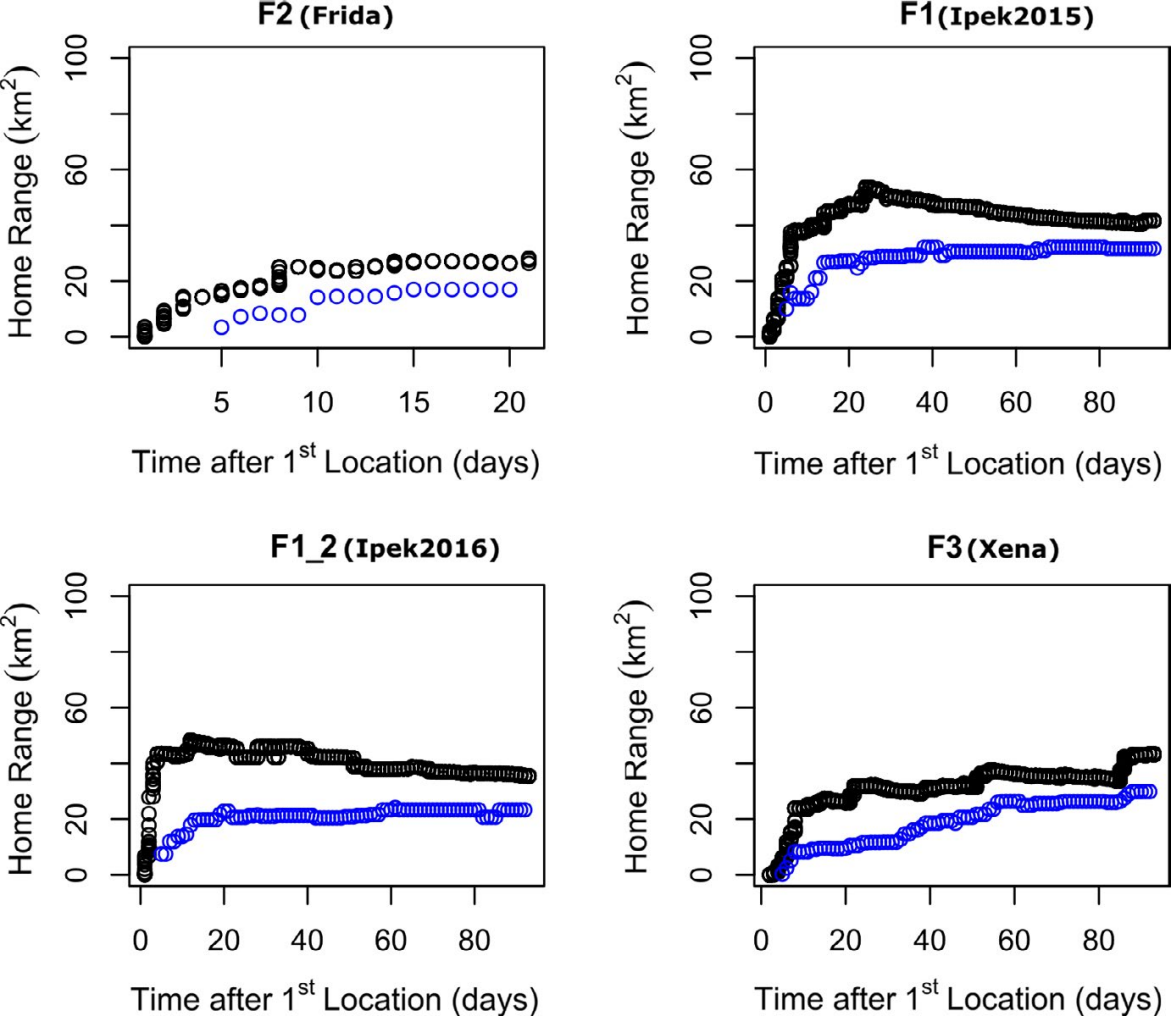
Comparison of cumulative MCP home range of the breeding territorial female Eurasian lynx *Lynx lynx* with 21 days tracking data to three months tracking data of breeding and nonbreeding territorial females. Black indicates all collected GPS locations, and blue indicates one random GPS location per day

**FIGURE 4:**
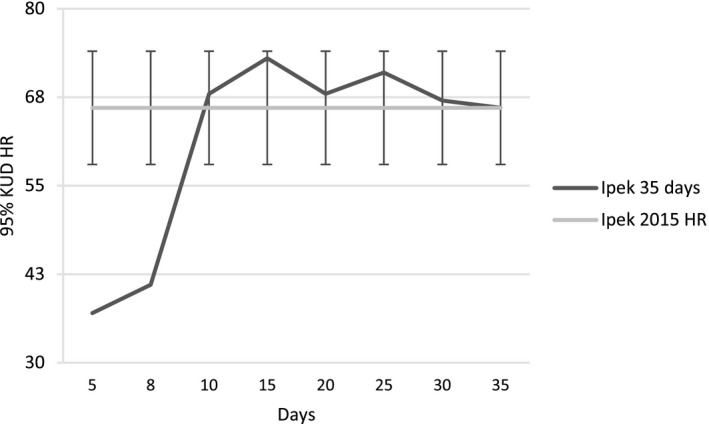
Home range size of adult territorial female Eurasian lynx *Lynx lynx* F1 during the first 35 days after capture and collaring in comparison with its mean home range size in 2015 (66.0 km^2^) excluding the summer season. Error bars indicate standard deviation for seasonal home range size (8.0 km^2^) excluding the summer of 2015

Two cumulative MCP HRs of the territorial breeding female F1 (F1, F1_2) reached asymptotic values between 15 and 20 tracking days (Figure 3). The cumulative MCP HRs of F2 with 21 tracking days and 276 GPS fixes (13.1 GPS fixes/day) also did not change between 15 and 20 days. Cumulative MCP HRs of the nonbreeding young female (F3) did increase with time (Figure 3). The Kernel HR size of breeding female F1 reached the asymptotic value of its annual HR size of 66.0 ± 8.0 km^2^ (obtained from autumn, winter, and spring HRs excluding the summer HR when she gave birth and used half of her 2015 HR) within 10 days. After this date, HR size fluctuated within one standard deviation at 68.0 ± 2.6 km^2^ during the following 25 days (Figure 4). We concluded that 15–20 tracking days were sufficient to estimate the MCP and Kernel HR of adult breeding females, which allowed us to use HR data of F2 to calculate a mean HR size for adult female lynx in our study area.

Females had the smallest mean Eurasian lynx HRs (*n* = 4, 95% KUD = 57 ± 26 km^2^, 95% MCP = 56 ± 32 km^2^, Figure 2, Tables 2 and 4) in the population. Mean HR size of territorial males (*n* = 2, 95% KUD = 176 ± 3 km^2^, 95% MCP = 183 ± 5 km^2^) was more than three times larger than female HRs (Table 2). The core areas (50%) of female HRs were very similar to each other with a mean of 15 ± 3 km^2^. HRs of the two adult male lynx M3 and M5 (floaters) were large (95% KUD = 2,419 ± 2,208 km^2^, 95% MCP = 1,888 ± 1,675 km^2^). M5 had 95% KUD and MCP HRs 70 and 55 times larger, respectively, than the female HRs (Figure 2, Table 2). In the first year of tracking, M3 used 95% KUD and MCP HRs equivalent to 15 and 13 times the size of the 95% female HRs, respectively. These were also five times larger than the subsequent HR as a territorial (95% KUD = 174 km^2^, 95% MCP = 145 km^2^) during the second year of tracking (Figure 2). Floater adult males held larger mean HRs than adult males with defended territories (Figure 2), at a mean of 14 times larger than territorial male KUD and 13 times larger than territorial male MCP HRs (Table 2).

We tracked one juvenile disperser male lynx (M1) for almost a year (346 days) until 22 months of age. Twenty days after being captured and collared together with his mother, M1 separated from F1 on 10 April 2015. During the following months, he moved across larger areas than his natal HR, covering up to 447 km^2^ 95% KUD or 713 km^2^ 95% MCP HR. At the end of the tracking period, he was still not a territorial. Juvenile male M4 was last encountered on the 18th tracking day (8 April 2016) still in his natal HR (95% KUD = 57 km^2^, 95% MCP = 38 km^2^). During subsequent data downloading field surveys, we could not locate him or receive data from his collar. Subadult male M6 stayed in the study area for a short time period and used a temporary HR of 95% KUD = 104 km^2^ and 95% MCP = 66 km^2^. Data download after the 21st day was not possible for it.

### 3.3 Lynx density

The closure test results (z = −0.003, *p* = .5) confirmed population closure. Camera traps were active during 1,391 camera trap days (ctd), and effective camera trapping effort was calculated as 81%. Twelve different adult individuals, seven males and five females, of lynx were photographed 36 times at 11 camera trap stations. Seven of these lynx, three males and four females, were repeatedly photographed 24 times, and five lynx were photographed once. We obtained 11 spatial recaptures (recaptures of the same individual at additional camera trap stations) for three male and three female lynx. Females were spatially recaptured at most at one, and males were spatially recaptured at four, three, and one stations.

The exponential detection function was the best fit (AICc = 317.70, log likelihood = −154.53) for the dataset when compared to half‐normal (AICc = 329.20, log likelihood = −160.27) and was therefore used to run the three density models. Examination of the AIC_c_ values identified the ‘behavior’ b_1_ model as the best fit. This produced a density estimate of 4.9 ± 1.6 (*SEM*) lynx per 100 km^2^ (95% confidence interval [CI] 2.7–9.1, Table 3). The capture probability at the center of the HR, *g_0_*, was estimated to be 0.17 ± 0.06 (*SEM*, 95% CI 0.08–0.32), and б estimated to be 986.6 ± 333.06 (*SEM*, 95% CI 517.4–1,880.2).

**TABLE 3:** Summary of model fit for spatial capture–recapture models in estimating Eurasian lynx population density in northwest Anatolia

Model	Notation	AIC_c_	ΔAIC_c_	AIC_c_ wt	log likelihood	K
Behavior	(*λ_0_* ~ b1, *б* ~ 1)	305.12	0.00	0.68	−145.70	4
Null	(*λ_0_* ~ 1, *б* ~ 1)	306.81	1.69	0.29	−150.08	3
Learnt response	(*λ_0_* ~ b2, *б* ~ b)	311.05	5.93	0.03	−154.51	5

Note

AIC_c_ is Akaike's information criterion adjusted for small samples sizes, **Δ**AIC_c_ is the difference between the smallest AIC_c_ value and all the others. AICc wt is the AICc weight, the conditional probabilities for each model. The model with the highest AIC_c_ wt is then the one with the highest support. K: number of parameters in the model.

### 3.4 Population density versus home range size

**FIGURE 5:**
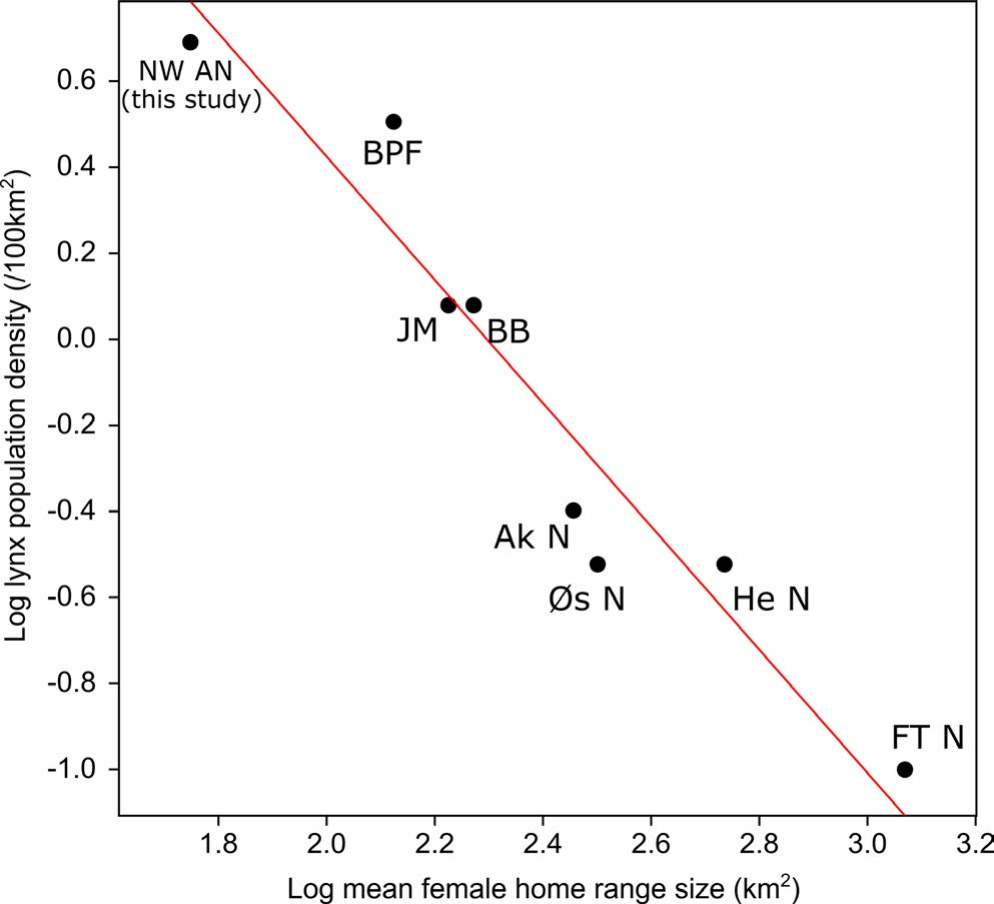
Relationship between mean female home range size and population density (both log transformed) of eight Eurasian lynx *Lynx lynx* populations. NW AN, Northwest Anatolia (this study); BPF, Biolawieza Primeval Forest (Jedrzejewski et al., 1996; Schmidt et al., 1997); JM, Jura Mountains (Breitenmoser et al., 1993; Zimmermann & Breitenmoser, 2007); BB, Bohemian‐Bavarian (Magg et al., 2016); Ak N, Akershus Norway; Øs N, Øsafjells Norway; He N, Hedmark Norway; FT N, Finnmark‐Troms Norway (Gervasi et al., 2013)

When compared to the Eurasian lynx populations from southern to northern latitudes, the lynx population in northwest Anatolia had the smallest mean female HR size and highest population density, and the lynx population in Finnmark‐Troms had the largest mean female HR size and lowest density (Table 4). In central Europe, the lynx population in Białowieża Primeval Forest (BPF) had the highest density and smallest mean female HR size when compared to other central European Eurasian lynx populations (Table 4). There was a strong, negative relationship between female HR size and population density (Spearman's ρ = −0.98, *p* < .01). Figure 5 shows the linear relationship between log‐transformed mean female HR size and lynx population density for eight Eurasian lynx populations.

**TABLE 4:** Mean 100% MCP female Eurasian lynx *Lynx lynx* home range size and population density at eight study areas distributed along a south–north latitudinal gradient

Population	HR [km^2^]	Lynx density [/100 km^2^]	References
NW Anatolia	56^1^	4.9^1^	1. This study
Jura	168^2^	1.2^3^	2. Breitenmoser et al. (1993) 3. Zimmermann & Breitenmoser, (2007)
Bavaria‐Bohemia	187^4^	1.2^4^	4. Magg et al. (2016)
Białowieża	133^5^	3.2^6^	5. Jedrzejewski et al. (1996) 6. Schmidt et al. (1997)
Akershus	286^7^	0.4^7^	7. Gervasi et al. (2013)
Østafjells	317^7^	0.3^7^	7. Gervasi et al. (2013)
Hedmark	544^7^	0.3^7^	7. Gervasi et al. (2013)
Finnmark‐Troms	1173^7^	0.1^7^	7. Gervasi et al. (2013)

Note

Numbers in the superscript indicate the reference study.

## 4 DISCUSSION

In this study, we report for the first time HR sizes and spatial behavior of Eurasian lynx from Southwest Asia using high frequency GPS tracking data, focusing on the potentially isolated yet unexploited northwest Anatolian population. As it is a high‐density lynx population, our results provide crucial information on spatial ecology and behavior of a felid population within a natural state. Our results are consistent with the high population density hypothesis and indicate that small territorial and strikingly large floater HRs result from high population density and competition for space. Our results are inconsistent with the prediction of the primary production hypothesis, since our study area is located in a region of low productivity and lynx occurred here at high densities and small HRs in contrast to the expectation of low population density and large HRs.

### 4.1 Daily distance moved by a lagomorph specialist lynx

The adult male and female individuals of our Eurasian lynx population had significantly lower mean body masses than adult male and female Eurasian lynx in Europe and northern Asia (Breitenmoser et al., 1993; Jedrzejewski et al., 1996; Sedalishchev et al., 2014). This might be an evolutionarily adaptive adjustment as in other lagomorph specialists such as the Canada (*L. canadensis*) and the Iberian lynx (*L. pardinus*). The daily distance moved (DDM) by our individuals should be directly influenced by this special diet because after successful kills they spent less time‐consuming their lagomorph prey (1 or 2 days; Mengüllüoğlu et al., 2018) and started to roam much earlier than lynx individuals in central and eastern Europe (Linnell et al., 2008). In a study in BPF, lynx doubled their straight‐line movements during periods of low prey availability (Schmidt, 2008). At low hare densities, this might mean longer searching times and distances. It is also possible that regardless of their territorial status, male lynx should cover larger distances than females in BPF in order to satisfy the physiological requirements of a larger body size (Jedrzejewski et al., 2002). In our study, DDM of individual lynx were very similar to each other regardless of their sex and territorial status: DDM of floater males with huge HR were similar to DDM of territorial males or females. These findings might indicate a sufficient prey base in our study area during the lynx tracking periods between 2015 and 2017. In the case of a sharp decrease in hare numbers, we might expect that this high‐density lynx population would have to increase their DDM (Schmidt, 2008), thereby increasing daily energy needs and the required daily food intake. For territorial females with small HRs, this could mean starvation and consecutive loss of kittens, leading to reduced recruitment and HR abandonment (e.g., *L. canadensis*; Poole, 1994), because increasing HR size is unlikely in a space completely occupied by neighboring territorial females.

### 4.2 Home ranges

Our study revealed the smallest adult female mean HR size for Eurasian lynx, a size comparable to HR size of the hare specialist Canadian lynx (mean female 100% MCP = 56 ± 23 km^2^, mean male 100% MCP = 134 ± 13 km^2^; Burdett et al., 2007). Although we collected only 21 days of tracking data (N_GPS_ = 276) for territorial breeding female F2, high frequency GPS fixes (14/day) enabled us to assess the HR size of this female and use it for mean female HR calculations. Most of the early Eurasian lynx HR studies also used similar numbers of tracking days and GPS locations for seasonal and annual home range assessments (e.g., minimums of 14 days per lynx for seasonal HRs and 40 locations for annual HR estimation: Schmidt et al., 1997; minimums of 14 locations for seasonal and 20 locations for annual HRs: Linnell et al., 2001). In 21 tracking days, F2 used almost all the area available to her as her HR was bounded by HRs of F1 in the south and F3 in the north (Figure 2), HR of another territorial female lynx in the west (Mengüllüoğlu et al., 2019), and a deep valley cut by a river in the east. Although the 95% HRs of the female lynx in our study area were somewhat variable, their core areas (50%) were almost the same size (Table 2).

The larger 95% HR of nonbreeding female F3 was a consequence of several exploratory movements to territories of neighboring females with kittens in September, one of which was her mother's territory (Mengüllüoğlu et al., 2019). This young female stopped exploratory movements during the last 40 days (October–November 2017) of her tracking period (29 March–25 November 2017) and used a 100% MCP HR of 60 km^2^ and a 95% KUD HR of 68 km^2^, very similar to the HR size before she started exploratory movements. We did not observe this type of straight‐line movements in adult breeding females and never encountered females with kittens in the core areas of other breeding females during camera trapping surveys between 2009 and 2018. Therefore, F3’s HR most probably settled toward the end of the tracking year, when neighboring breeding females started to enlarge their seasonal HRs at the beginning of winter (Schmidt et al., 1997).

Mean adult territorial male HRs were smaller than in central and southern Europe, such as populations in Jura Mountains (e.g., 100% MCP = 283 km^2^; Breitenmoser‐Würsten et al., 2007), BPF (e.g., 100% MCP = 248 km^2^; Schmidt et al., 1997), and Macedonia (e.g., 100% = 466 km^2^; Melovski et al., 2020). In general, male territories are known to overlap with one to two female HRs in Eurasian lynx populations (Schmidt et al., 1997). In our study, area territorial male HRs can overlap with three to four female HRs and adult floater male HRs can overlap with up to 70 female HRs (Table 2).

Besides small territorial HRs, our study also revealed nonterritorial adult male lynx with strikingly large HRs. These ‘floater’ lynx were both 8–10 years old; therefore, they were not young dispersers. They had very large HRs in comparison with other Eurasian lynx, one of them with a HR size comparable to HR sizes of male lynx in far northern latitudes such as Scandinavia (Herfindal et al., 2005; Linnell et al., 2001). The other floater male had one of the record size HRs (almost 4,000 km^2^) for an adult Eurasian lynx (Linnell et al., 2021). Dispersing young male individuals are known to range over larger areas than territorial adult males (Schmidt, 1998). Our adult floater males had stable HRs, visiting the same places repeatedly. To our knowledge, floater adult Eurasian lynx older than the age of four and with such large stable HRs have never been recorded by any previous study. We suggest that the high lynx density in our study area and a landscape fully occupied by adult territorial male lynx (breeding lynx populations: Mengüllüoğlu, 2010; Akbaba & Ayas, 2012, 2017; Breitenmoser et al., 2015; Mengüllüoğlu et al., 2015, 2019; Soyumert, 2020; Soyumert et al., 2019; Turan, 1984) has led to the presence of adult floater males queuing for territories (cf. Melzheimer et al., 2018). This is consistent with the observation that one of these adult floater males (M3) shrank his floater HR to one‐fifth of its previous size and apparently established a small permanent territory during the second year of tracking.

Floaters in our study might have been territorials before and/or waited for some time to establish a territory after they separated from their mothers. Our data do not allow us to assess this since they were not encountered on camera trap pictures during previous study years (since 2009) before their capture and collaring. This was in contrast to the only territorial adult male during his capture (M2), monitored by camera traps since 2012 and still a territorial in summer 2018 even after the battery of his collar failed. Other adult territorial male lynx, some of which escaped from the live traps during our live capture survey, also held long‐term territories and were camera‐trapped frequently since 2009 (8, 6, and 3 years; Mengüllüoğlu et al., 2019). All four territorial males displayed defensive marking behavior such as frequent cheek rubbing, claw marking, and fecal scrape marking, which were genetically identified to belong to them (long‐term monitored male territorial lynx in Mengüllüoğlu et al., 2015, 2019).

Interestingly, the floater with a striking HR size (M5) did not leave the close proximity of the live trapping location after capture and collaring and stayed in the study area for almost 1 month during the mating season, visiting three adult female territories. Therefore, a large HR might help floaters to search for vacant male territories and also assist them in seeking out females during the mating season.

### 4.3 Density

Our density estimate indicated one of the highest adult lynx densities ever reported for Eurasian lynx (4.9 lynx/100 km^2^), higher than the density of 4.2 independent lynx/100 km^2^ for a lynx population in southern Turkey (Avgan et al., 2014), based on the “behavior model” for estimating density. Besides showing the best fit to the data, the “behavior model” is likely to reflect the reality in our study area as we used lynx capture–recapture data also from camera trap stations near live traps. Eight of 12 lynx individuals captured by camera traps during the CMR survey were caught in live traps in the 2014–2015 and 2015–2016 live trapping seasons; four of these eight individuals were collared. Live captured lynx did not visit the camera trap stations (*n* = 5) near the live traps again and were only captured by camera traps away from live traps, thereby reducing their capture probability (g_0_). This pattern was associated with a decrease in camera trap visitation rates of live captured territorial lynx and responsible for video and GPS data which showed occasions of close presence to camera traps without leading to pictures and videos of adults, only of kittens (DM unpublished data). Therefore, the scale of movement (б), a parameter of the “learnt response” model, was not affected—we only observed a decrease in capture rates of live trapped individuals.

Lynx populations in our study area and in southern Turkey occurred at high densities were lagomorph hunters and lived in sympatry with high‐density lagomorph prey. Cannibalism and marking behavior such as scraping were previously also reported for this lynx population and are possibly a consequence of high lynx density (Mengüllüoğlu et al., 2018). Our study revealed significantly smaller body sizes for our adult lynx in comparison with lynx in Europe. Also, lynx in Anatolia consume 50% (900 g/day) of the food intake of lynx populations in Europe (1,800–2,000 g/day; Mengüllüoğlu et al., 2018). It is likely that hare densities in northwestern and southern Anatolia (NW Anatolia, 88 hares/km^2^; S Anatolia, 36 hares/km^2^; Mengüllüoğlu et al., 2018) are therefore sufficient to sustain high‐density lynx populations. The relationship between hare population dynamics and lynx numbers should be investigated further, to assess this association in detail. This would require the monitoring of lynx and hare populations over the long term in major ecosystem types of Anatolia (Mengüllüoğlu et al., 2018). Another reason for high lynx density in Anatolia might be the absence of quota hunting and low levels of lynx poaching in major lynx habitats—Eurasian lynx were never mentioned to be a source of human–wildlife conflict in Turkey, consistent with its dietary preferences (Mengüllüoğlu et al., 2018).

### 4.4 Home range and density of Eurasian lynx populations

Our study documented the size of HRs of three territorial females through GPS tracking. From our camera trap photographs, we know that seven neighboring female territories occurred in our study area between 2009 and 2018. As our study area is surrounded by deep valleys and is topographically well defined, it is likely that this topography also defined the borders of female territories (Mengüllüoğlu et al., 2019). In this case, the presence of seven territories would give an average of 57 km^2^ per female HR, consistent with our GPS tracking results.

As in our study area, widely distributed mixed dry coniferous and steppe ecosystem in Anatolia receives much lower mean annual precipitation, experiences dry, and hot summers and has a lower primary productivity than deciduous and mixed temperate forest ecosystems in Europe and at the Black Sea coast of Turkey (Evrendilek et al., 2007). Despite this lower primary productivity, Eurasian lynx in Anatolian ecosystems occurred at the highest densities (this study and Avgan et al., 2014) and smallest HRs. This is not consistent with the view that primary productivity might be the main driver of HR size in Eurasian lynx. Moreover, low primary production does not always lead to low prey density, because locally adapted prey species can persist at high population densities at low productivity and dry climatic regions (Baghci et al., 2003; van Duyne et al., 2009; Kaplan, 1995; Mengüllüoğlu et al., 2018). Therefore, primary production cannot fully explain the variation in Eurasian lynx HR size throughout its global distribution (Europe and Asia). Our review of the literature demonstrates a strong negative correlation between lynx density and HR size (Figure 5). Similarly, in populations of many solitary territorial species (Lindeman et al., 2015; Šálek et al., 2015; Wiens et al., 1985; Wood et al., 2012) and in Scandinavian Eurasian lynx, individual HR size decreased with increasing conspecific density as long as prey availability was sufficient (Aronsson et al., 2016).

Perhaps, if the prey base is not limited (as in the case of seasonal migration of reindeer in northern Scandinavia: Matisson et al., 2014), the low density and large HR size of lynx in many central European ecosystems might be the consequence of anthropogenic factors such as harvesting or poaching of many lynx individuals (Aronsson et al., 2016; Heurich et al., 2018; Nilsen et al., 2012; Šálek et al., 2015). It is noteworthy that in our study area adult territorials had very small HRs, yet floater males had enormous HRs, covering up to 70 female HRs. This suggests that HR size might be first and foremost a direct result of population density and competition for (breeding) space among individuals of carnivore populations as long as prey density and biomass are sufficient (Aronsson et al., 2016; Lindeman et al., 2015; Šálek et al., 2015; Wiens et al., 1985; Wood et al., 2012).

## 5 CONSERVATION IMPLICATIONS

The results of our study provide crucial information for Eurasian lynx ecology and behavior as it reveals what we consider to be natural processes in the spatial organization of unexploited and high‐density felid populations. Competition for space and a landscape fully occupied by adult territorial individuals may lead to small territorial HRs and delay territory establishment in males. As a result, adult floater individuals may roam across very large HRs while queuing to take over vacant territories (Melzheimer et al., 2018, 2020). Our population constitutes a good model for understanding the evolutionary behavior and dynamics in Eurasian lynx populations. It seems that, regardless of ecosystem productivity, lynx populations can occur at high densities as long as the locally adapted prey base is maintained. Perhaps the well‐protected lynx and prey populations in Białowieża Primeval Forest were a good example of this, as this lynx population was at one of the highest densities in Europe. We recommend the use of data from lynx populations in natural states (BPF, northwest Anatolia) for the purpose of modeling reintroduction scenarios and carrying capacities for Eurasian lynx in Europe. Conclusions derived from exploited or reintroduced lynx populations might not reflect evolved behavior of Eurasian lynx populations and result in misleading assumptions. We also recommend preservation of the current lynx habitats and long‐term lynx and prey population monitoring in northwest Anatolia to understand the long‐term dynamics and interactions in this lynx population.

## CONFLICT OF INTEREST

The authors have no competing interests.

## AUTHOR CONTRIBUTION

**Deniz Mengüllüoğlu:** Conceptualization (lead); Data curation (lead); Formal analysis (equal); Funding acquisition (equal); Investigation (lead); Methodology (lead); Project administration (lead); Resources (equal); Validation (equal); Visualization (lead); Writing‐original draft (lead); Writing‐review & editing (lead). **Sarah Edwards:** Formal analysis (equal); Methodology (equal); Writing‐original draft (supporting); Writing‐review & editing (supporting). **Heribert Hofer:** Formal analysis (equal); Funding acquisition (equal); Project administration (supporting); Resources (equal); Supervision (lead); Validation (equal); Writing‐review & editing (equal). **Anne Berger:** Formal analysis (equal); Funding acquisition (equal); Methodology (equal); Project administration (supporting); Resources (equal); Supervision (supporting); Validation (equal); Writing‐review & editing (equal).

## Data Availability

The data that support the findings of this study are available upon reasonable request from the corresponding author (D.M.). The data are not publicly available because of the conservation status of the species and the currently growing issue of illegal hunting that it may face in parts of the country.
